# Disease of the Antrum

**Published:** 1850-10

**Authors:** 


### ARTICLE XIV.

### *Disease of the Antrum.*

The diagnosis between enlargement of the antrum by a morbid growth within its cavity, and the gradual expansion of the walls of that sinus by fluid, requires, according to the records of surgery, a great deal of care. The cases where the distinction presented some difficulty are doubtless of considerable value; we therefore think we are rendering good service to our readers by giving them the outline of a very interesting case of that description lately under the care of Mr. Fergusson, of King's College Hospital.

*History.*—Marianne S., twenty-six years of age, was admitted into the Adelaide ward, March 23, 1850; was born in Hampshire, and has always lived in the country. Her health has never been strong; she has always been looked upon as delicate, and is subject to severe headaches. Patient has been married eight years, and the youngest of her four children is five months old.

About eight months ago, a swelling appeared on the right cheek, in the situation of the antrum, about three-quarters of an inch external to the right ala nasi, and on a level with that process. It came on imperceptibly, without any pain, and when she first noticed the tumor it was about the size of a pea, immovable, and not painful on pressure. Since that time it has gradually increased, but of late somewhat rapidly.

On admission, the tumor was about the size of a hen's egg, involving the whole of the non-alveolar portion of superior maxilla on the right side. Externally, it formed a large, smooth, undiscolored projection, drawing the eye a little downwards. On passing the finger inside the cheek and along the outside of the teeth, the quantity of gum between the latter and the projection of the swelling was felt to be very small. On looking into the mouth, the right half of the hard palate presented, instead of the normal concavity, a smooth convexity, rather more than an inch long, and half that width, this convexity lying between the median line and the gum of the molars and bicuspids of that side. On pressure, which gives the patient no pain, there is a feeling of some fluctuation, and the hand experiences an elastic tense sensation. When strong pressure is made on the cheek, the patient feels the teeth thrust downwards.

The first and third molar, and the first bicuspid, are destroyed by caries, and the other teeth are likewise affected; in fact, the patient has always suffered from tooth-ache, and she has hardly a sound tooth in her head.* The zygomatici muscles have been so stretched, that they have become paralyzed, and on smiling, the right corner of the mouth, instead of being drawn upwards, is pulled downwards and outwards, the triangularis oris muscle alone acting. The most yielding part of the tumor is the situation where it first appeared. Five days after admission, the patient was attacked with severe inflammations on the *left* side of the face, involving the glands and gums. She was some time before she recovered from this complication, the disease of the antrum remained, however, in the same state as before described.

Mr. Fergusson, considering that the time had now arrived to adopt operative measures for this affection, had the patient brought into the theatre on the 27th of April, and determined to proceed to the removal of the superior maxilla, if the cavity of the antrum, where really, as was suspected, found occupied by a solid tumor. Bearing, however, in mind that the appear-

* Bordenave says, as quoted by Mr. Stanley, "Caries of the teeth is the cause of almost all the diseases of the antrum and neighboring parts."

ances presented *might* be owing to an accumulation of fluid, the operator resolved to make an exploring puncture through the gum, before any further steps were adopted. An exploring needle was therefore passed into the tumor, and when fluid had been seen to escape, Mr. Fergusson took up the scalpel, and made an incision through the gum (the patient having been rendered insensible by chloroform) down to the anterior wall of the antrum, and took out a portion of the same, which was much thinned. By this proceeding, a quantity of glairy fluid containing flakes of cholesterine, was let out of the antrum, and the nature of the tumor being thus ascertained, no further interference was required, and the patient removed. The swelling went on diminishing in size for the next few weeks, and the patient was discharged on the 3rd of May, in a pretty satisfactory condition. Mr. Fergusson performed an operation of a somewhat similar character as the above, on the 22nd of June last. Here the patient was a man fifty years of age, who stated, that about seven years ago he was much troubled with the tooth-ache on the right side of the superior maxilla, and he noticed that that bone was increasing in size. This went on until about four years ago, when the tumor was lanced through the gum, and about half an ounce of thick, reddish fluid like blood escaped. Though the swelling decreased a little, the improvement did not last long, and the tumor again increased, and discharged pus from the alveolus. On admission, the patient presented a hard, osseous tumor, projecting from the superior maxilla, about the size of a pigeon's egg. In the mouth, a hard but yielding portion was felt to extend along the outer side of the alveolar process ; and was obviously hollow. The patient had never experienced any pain in the tumor. The operator made a longitudinal incision into the right nostril, and reflecting the flap upwards and outwards, removed the anterior wall of the antrum with the curved bone forceps.

After the operation, Mr. Fergusson stated that the pupils would doubtless take unusual interest in the case, in consequence of what he had said at his last clinical lecture; as also from the circumstance that an instance of a somewhat similar kind had so recently been under notice in the hospital. The case just operated upon was in many respects similar to that of the female who had left the house a few weeks ago. There was one important difference—viz. that it was of older date. The swelling here had been present about eight years, and had varied in size at different times. One thing was particularly worthy of notice; that considerable deformity—in the shape of a large tumor on the cheek—remained. The discharge of the contents of the antrum had not permitted the expanded walls to resume their natural condition; and time alone could show whether such a desirable result would follow what he had done for the female patient. When he had last heard of her she was in excellent health; but it might be doubted if the swelling would ever entirely subside.

The man on whom he had just operated was anxious to have the conspicuous tumor removed; and Mr. Fergusson had therefore cut away the front wall of the antrum, which measure, as would be perceived, had the desired effect. He had, on a former occasion, observed, that such a condition as this, although well known to surgeons, was nevertheless not met with by any means so frequently as solid growths within the antrum, or connected with the jaw-bone.

Here (showing a cast) was an illustration of a remarkable case of this description. This was a cast which he had taken from the head of an old woman, who had an enormous enlargement on the side of the face, which had burst some few days before death. It would be observed what a large horizontal projection there was immediately under the orbit, and how a plate of bone projected forwards from the alveolar ridge, both of which produced great deformity. In this instance, the swelling had been larger than in the two cases which had recently been under treatment. The plates of bone were sufficient to show that the deformity was likely to remain, unless removed by such a proceeding as that which he had just executed.

Mr. Fergusson then concluded his observations by referring to various drawings and casts from cases which he had formerly had under his care, and dwelt particularly upon the various incisions on the face which surgeons had made in dealing with tumors in this locality, adverting especially to the division of the upper lip in the mesial line—the incision at its upper end running into the nostril on the affected side—a plan of operation which he considered peculiarly his own, and which he thought was attended with less inconvenience during the operation, and followed by less deformity at a subsequent date than any with which he was acquainted.—*London Lancet.*

